# Microscopic assessment of the sealing ability of three endodontic filling techniques

**DOI:** 10.4317/jced.52847

**Published:** 2016-02-01

**Authors:** Roger Cueva-Goig, Leopoldo Forner-Navarro, Mª Carmen Llena-Puy

**Affiliations:** 1 Associate Professor. Department of Stomatology, Universitat de València, Spain; 2 Professor. Department of Stomatology, Universitat de València, Spain

## Abstract

**Background:**

Several techniques have been proposed for root canal filling. New rotary files, with non-standardized taper, are appearing, so, points adapted to the taper of the last instrument used to prepare the canal can help in the obturation process. The aim of this study is to assess the sealing ability of different root canal filling techniques.

**Material and Methods:**

Root canals from 30 teeth were shaped with Mtwo and divided in three groups; A, standard lateral condensation with size 35 and 20 gutta-percha points; B, standard lateral condensation and injected gutta-percha; C, single gutta-percha point (standardized 35 Mtwo), continuous wave technique and injected gutta-percha. Root surfaces were covered with nail varnish, except for the apical 2 mm, and submerged in a NO3Ag2 solution; apical stain penetration was measured in mm. Data were compared using the Kruskal-Wallis test with a 90% confidence interval.

**Results:**

A and B groups showed stain leakage in the 90% of the cases, whereas it was of 80% for group C. Stain leakage intervals were 1-5 mm for groups A and B and 1-3 mm for group C. There were no statistically significant differences between the three studied groups (*p*>.05).

**Conclusions:**

All the analyzed root canal filling techniques showed some apical stain leakage, without significant differences among them.

** Key words:**Gutta-percha filling, microleakage, single cone, injected gutta-percha, warm gutta-percha.

## Introduction

Tridimensional filling of root canal system is an essential phase in the canal treatment. Its aim is to achieve a filling of the canal system with the possible best sealing, in order to aisle it from the outside (1). Several materials and techniques have been proposed and investigated for root filling, but none of them fulfills all the desirable requirements (2).

The introduction of rotary instrument systems for root canal preparation has been parallel to the development of obturation techniques, in order to achieve more effective and simple clinical procedures. Apart from the classical technique (lateral condensation), other alternatives are proposed, such as continuous wave obturation (3,4), injected gutta-percha (5,6), mixed techniques, or the use of softened gutta-perchas introduced in the canal through a carrier (7-9). Tapered points adapted to the conicity of the last instrument used to prepare the canal, help in the obturation process (10,11).

In the present study we aim to assess the sealing ability of three canal filling techniques (lateral condensation, lateral condensation with injected gutta-percha and the use of single-cone technique with a gutta-percha point of adapted tapered followed by continuous wave and gutta-percha injection). Our null hypothesis is that new obturation techniques possess a similar sealing ability than the classical lateral condensation technique.

## Material and Methods

-Sample size: After getting the authorization from the Universitat de València Ethics Committee, 40 extracted human teeth were used. They were randomly assigned to 3 experimental groups (ni=10): A, B and C, and two positive and negative control groups, D and E (ni=5). Inclusion criteria were: recently extracted and hydrated human teeth (incisors, canines and palatal roots of maxillary molars), with straight roots, internal morphology Wein type I (only one root canal), and the possibility of canal permeabiliza-tion with a number 10 K file.

-Sample preparation: Immediately after extraction, teeth were stored in saline with 0.1 % thymol. Anatomic crowns were detached from roots with a thin diamond drill -FG D16/6 CB- (Intensiv, SA, Grancia, Switzerland). Disinfection and organic debris elimination was carried out through an immersion in 2 % sodium hypochlorite for 60 minutes. The access to the root canal was prepared through a diamond spherical drill for cameral opening FG 201 NL/6 (Intensiv, SA, Grancia, Switzerland).

Root canal length was established with a number 10 K-file (Dentsply Maillefer, Ballaigues, Switzerland), until the tip was visible through the apical foramen, and then subtracting 1 mm. Root canals were prepared by the same operator with the nickel-titanium rotary file system Mtwo, to number 35, with .04 taper ( Table 1), using a VDW SILVER motor (ATR Srl, Pistoia, Italy) and a Sirona contra-angled handpiece with a 6:1 reduction (Sirona Dental Systems GmbH, Bensheim, Germany). Irrigation was carried out after each file change, through an endodontic irrigation syringe with blunt Monoject tip (Sherwood Medical, St. Louis, USA), containing 5 ml of 2.5 % sodium hypochlorite at 60 ºC and 2 ml of EDTA with urea peroxide (Glyde File Prep EDTA gel, Dentsply-Maillefer, Ballaigues, Switzerland). The final irrigation was performed with 5 ml of 2.5 % NaOCl at 60 ºC. After that, canals were irrigated with sterile water, dried with number 35 ISO paper points (Dentsply-Maillefer, Ballaigues, Switzerland), and apically permeabilized with a number 10 K-file (Dentsply-Maillefer, Ballaigues, Switzerland). Teeth were stored in a saline wet gauze, until their filling.

**Table 1:**
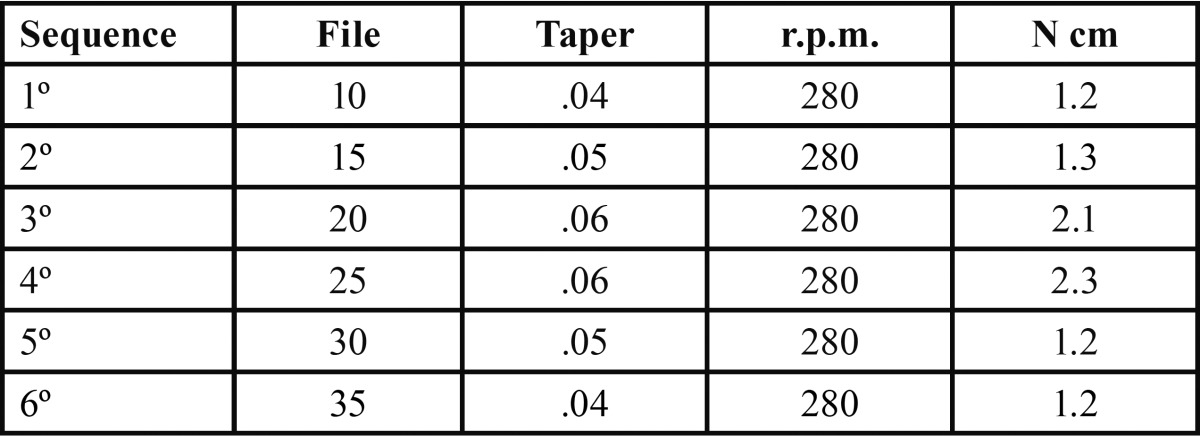
Mtwo sequence.

-Study groups:

Group A: standard lateral condensation obturation, with a gutta-percha point ISO number 35 (Dentsply-Maillefer, Ballaigues, Switzerland), lateral condensation through a number 25 digital spacer (Dentsply-Maillefer, Ballaigues, Switzerland) with number 20 ISO accessory points (Dentsply-Maillefer, Ballaigues, Switzerland). Group B: mixed technique of standard lateral condensa-tion and injected gutta-percha through Beefill system (VDW® GmbH, München, Germany), introduced through the space created by the digital spacer.

Group C: single gutta-percha cone Mtwo (Dentsply-Maillefer, Ballaigues, Switzerland), continuous wave technique with System B Heatsource (EIE/Analytic Technology, CA, USA) and injected gutta-percha obturation (Beefill, VDW® GmbH, München, Germany).

Group D: positive control, without obturation.

Group E: negative control, obturation and total impermeabilization with cyanoacrylate peripheral sealing (Loctite Super Glue 3, Henkel Ibérica SA, Barcelona, Spain). An epoxy resin cement (TopSeal, Dentsply-Maillefer, Ballaigues, Switzerland) was introduced with a number 001 25 mm lentulo (Dentsply-Maillefer, Ballaigues, Switzerland). The gutta-percha was compacted with a digital instrument (Dentsply-Maillefer, Ballaigues, Switzerland). After the obturation, the cement was let to set at room temperature for 7 days. After that, all 5 groups were first brushed with a layer of cyanoacrylate (Loctite Super Glue 3, Henkel Ibérica S.A., Barcelona, Spain), and then with two layers of nail varnish, except for the 2 apical mm. A different color was used for each experimental group.

-Staining: Specimens were introduced in opaque containers with 50 % silver nitrate for 4 hours. After that, they were washed with water for 30 minutes and dried at room temperature for 24 hours.

-Section preparation: Samples were included in methacrylate resin, in order to have a better handling, and were stored for 48 hours at stable temperature until their total polymerization. Resin included material was progressively worn down perpendicularly to the root axis, with a polisher, Grinder/Polisher 900 (SBT South Bay Technology INC, San Clemente, USA). After each millimeter progression, the presence or absence of dyer was registered. For that purpose, each obtained section was exposed to sunlight for 2 hours, to let the dyer out.

-Microscopic observation: Each section was observed with an stereomicroscope Opmi Pico (Carl Zeiss MicroImaging Inc., Thornwood, USA), with a 2.5X magnification. Images were captured with the program PCTV Vision 2.75 (Pinnacle Systems Inc, Mountain View, USA).

-Image assessment: Criteria for leakage assessment was the presence or absence of silver nitrate dyer, filtered through the apical foramen, in the interface between dentine and gutta-percha-cement, or between the gutta-percha points and the cement. Images were analyzed with the photographic program Adobe Photoshop Elements 6.0 (Adobe Systems Incorporated, San José, USA).

-Statistical analysis: It was performed through the Kruskall-Wallis non-parametric test, with the program SPSS 19 (SPSS Inc., Chicago, USA), and using a confidence interval of 95 % (*p*>0.05).

## .Results

Group A (lateral standard condensation) presented filtration in 9 out of 10 studied samples. Filtration intervals ranged from 1 to 5 mm, with an average of 2.0 mm.

The mixed filling technique (lateral condensation and injected gutta-percha) -group B- showed an average depth penetration of 1.7 mm, with an interval of 1 to 5 mm. In this case, the presence of dye was observed in 9 out of 10 sample group elements.

In group C (single cone, continuous wave and injected gutta-percha), dye filtration was present in 8 out of 10 studied samples. The filtration range was 1 to 3 mm, with an average of 1.40 mm.

**Figure 1:**
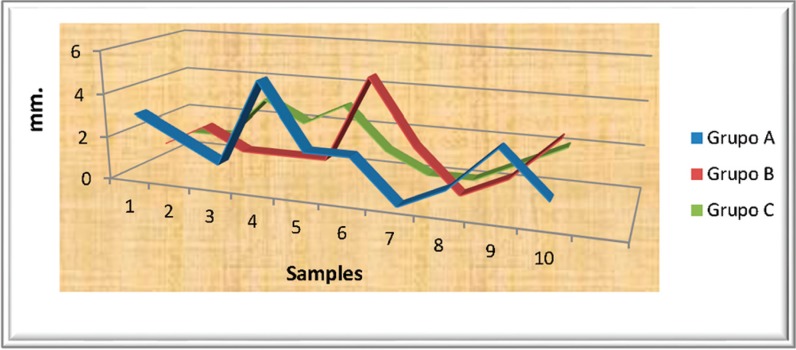
Results including the three study groups.

Groups A, B and C: with the filtration results, the average, standard deviation and confidence intervals for the average were cal-culated for each of the groups ( Table 2, Fig. 1).

**Table 2:**
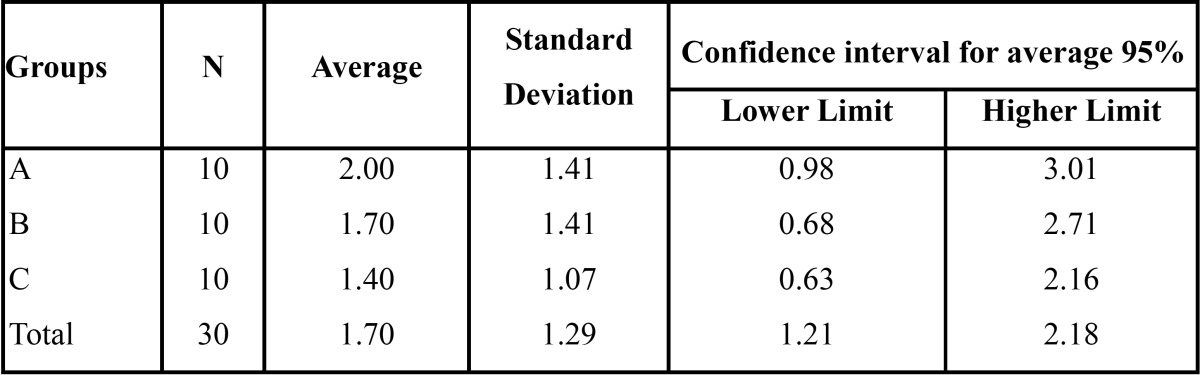
Data analysis.

No statistically significant differences between the three experimental groups were observed, referring to dye penetration levels, therefore, confirming our initial hypothesis.

## Discussion

A perfect obturation should be well condensed, seal all the canals connecting the pulp space with the periodontum, be adapted to the previous instrumented canal walls and end in the apical constriction (1,2). These assertions are yet a goal of canal treatment, due to the lack of total canal sealing, in all the published filtration studies, including the present one.

In our study groups, the one presenting less filtration was the one sealed with single cone gutta-percha, System B and injected gutta-percha, followed by the mixed technique and finally the sealed with lateral condensation, although with no statistically significant differences (*p*<0.05).

To determine gutta-percha sealing ability, several studies on bacterial filtration through root canals have been performed. Molander *et al.* (3) frequently found *Enterococcus faecalis* in sealed root canals. Similarly, in other longitudinal section studies, sealed teeth with an inadequate lateral sealing or obturation length, showed a higher frequency of periapical lesions, than teeth with a good endodontic treatment (4,5).

Cold gutta-percha lateral condensation is a worldwide extended technique, due to the advantages of controlled gutta-percha placing inside the root canal, and the low economic cost (6,7); although it is a time consuming procedure. Lateral condensation technique produces cold welding, a non uniform mass of gutta-percha points in the coronal, medium and apical thirds with no perfect replication of the canal, letting spaces filled with sealant (9). The final obturation comprises a great number of gutta-percha points firmly tightened, bound by friction and cement, like an homogeneous mass (2).

In some studies, similar to this work, single cone technique filtration resistance was compared to that of other obturation techniques. No differences in apical filtration were detected for sealed root canals using lateral condensation or single cone techniques (10). Similarly, no differences were found between single cone technique and vertical condensation (11). Single cone obturation was introduced in order to minimize the sealing component, through the use of gutta-percha points, that globally closed the geometry of nickel-titanium instrumentation systems (12). This technique is much more quick and easy. The treatment results of an *in vivo* study comparing single cone technique and lateral condensation, showed no significant differences, after a period of 6 to 18 months (13).

The use of System B HeatSource® offers a modification of the warm gutta-percha technique (14). The main advantage of the continuous wave condensation technique through this device, is that the gutta-percha apical filling can be performed through a continuous movement, with a plugger, electrically heated at the temperature recommended by the manufacturer (200 ºC) (15), therefore, allowing accessory canal filling (16).

In a meta-analysis, Peng *et al.* (17), concluded that gutta-percha has a higher risk of filling overextension that cold lateral condensation; these authors got similar results for postoperative pain prevalence, long term results and filling quality. These observations do not match the ones of the present work, were no case of gutta-percha overextension was detected; this could be due to the use of gutta-percha combined with a single standard cone, that would fill the apical foramen space. Venturi *et al.* (18) compared the ability of lateral canal filling of two gutta-percha techniques, achieving better filling rates with vertical condensation with apical filling, than with Schilder technique. Differing from our results, Collins *et al.* (19) got better results with gutta-percha techniques, in a comparison of three filling techniques, two with gutta-percha (lateral and vertical condensation) and one with cold lateral condensation. Brothman (20) showed that vertical condensation with gutta-percha sealed approximately twice as much lateral canals than gutta-percha lateral condensation. On the contrary, in our study, the gutta-percha sealed group, despite presenting less filtrated samples (not statistically significant), showed a higher filtration than the rest of groups. Gutta-percha vertical condensation, using the continuous wave technique, can increase the density of gutta-percha mass and homogeneity in previous cold lateral condensation sealing (21). In fact, in our study, group C presented higher gutta-percha mass and lower cement than with standard lateral condensation. This could be due to the fact that group C was sealed with a single gutta-percha cone Mtwo system (with the same taper of the last instrument used for canal preparation), and thus a good initial adaptation to the canal was achieved, bearing in mind that selected canals were not oval, but rather round. Later, when the single cone was heated with System B, it melted with the walls, and finally, the gutta-percha filled the remaining spaces. Our work matches that of other authors, that pointed out that this technique allows a better fluidity inside canal irregularities (22-24). Moreover, other authors also concluded that this technique allows the introduction of a gutta-percha homogeneous mass into the canal system, with the carrier as condenser (25), and could be more effective in lateral canal obturation, than cold gutta-percha lateral condensation (23).

Yilmaz *et al.* (26) compared the sealing ability of two vertical condensation, BeeFill 2 in 1 and System B/Obtura II, with that of single cone technique with cold lateral condensation. After a period of two weeks using the in vitro fluid conductance procedure, they found worse results with the vertical condensation techniques.

## Conclusions

The analyzed techniques (mixed –combination of lateral condensation and injected gutta-percha- and single cone, with the same taper as the last instrument preparing the canal, together with continuous wave technique and injected gutta-percha), presented the same sealing ability, measured with a dyer, than conventional lateral condensation technique. However, the single cone technique, was the one presenting the lower dyer penetration, followed by the mixed technique and the lateral condensation.
